# Spatio-Temporal Determinants of Dengue Epidemics in the Central Region of Burkina Faso

**DOI:** 10.3390/tropicalmed8110482

**Published:** 2023-10-25

**Authors:** Cheick Ahmed Ouattara, Tiandiogo Isidore Traore, Boukary Ouedraogo, Bry Sylla, Seydou Traore, Clement Ziemle Meda, Ibrahim Sangare, Leon Blaise G. Savadogo

**Affiliations:** 1 Doctoral School of Health Sciences, Nazi Boni University, Bobo-Dioulasso 1091, Burkina Faso; seyd3@yahoo.fr; 2 Higher Institute of Health Sciences, Nazi Boni University, Bobo-Dioulasso 1091, Burkina Faso; tiandiogo2002@yahoo.fr (T.I.T.); medacle1@yahoo.fr (C.Z.M.); babaibrasangare@yahoo.fr (I.S.); gueswende@hotmail.com (L.B.G.S.); 3 Directorate of Health Information Systems, Ministry of Health, Ouagadougou 7009, Burkina Faso; ouedbouks@gmail.com (B.O.); syllabry@gmail.com (B.S.)

**Keywords:** dengue, spatio-temporal analysis, climate, Burkina Faso, outbreak

## Abstract

The aim of this study was to analyze the spatio-temporal distribution and determinants of the 2017 dengue epidemic in Burkina Faso. A principal component analysis of meteorological and environmental factors was performed to reduce dimensions and avoid collinearities. An initial generalized additive model assessed the impact of the components derived from this analysis on dengue incidence. Dengue incidence increased mainly with relative humidity, precipitation, normalized difference vegetation index and minimum temperature with an 8-week lag. A Kulldoff Satscan scan was used to identify high-risk dengue clusters, and a second generalized additive model assessed the risk of a health area being at high risk according to land-use factors. The spatio-temporal distribution of dengue fever was heterogeneous and strongly correlated with meteorological factors. The rural communes of Sabaa and Koubri were the areas most at risk. This study provides useful information for planning targeted dengue control strategies in Burkina Faso.

## 1. Introduction

Dengue is the world’s most widespread arbovirus. It occurs in tropical and subtropical regions throughout the world with a predilection for urban and semi-urban areas. Today, dengue fever is a public health concern due to its soaring incidence, with almost 390 million cases and 20,000 deaths per year in 128 countries, and the appearance of epidemic outbreaks [1,2]. Humans are the main reservoir of the dengue virus, whose four serotypes are transmitted by the bite of infected females of Aedes mosquitoes (*Aedes aegypti* and *Aedes albopictus*) [1]. *Ae. aegypti* is the main vector of dengue fever in Africa, and its life cycle is influenced by climatic factors such as ambient temperature, relative humidity and rainfall. Higher temperatures promote larval development and shorten the incubation period of the virus, increasing the proportion of infected vectors and therefore the risk of transmission. Conversely, however, very high temperatures can also destroy mosquitoes. Rain can have a variety of effects: in large quantities, it can eliminate eggs and larvae from potential containers in the short term, but residual water can create breeding habitats in the long term [3,4,5,6,7]. In fact, *Ae. aegypti* inhabits a variety of containers, both natural (tree holes) and artificial (tires, discarded items and other water containers). A dry climate combined with insufficient sources of running water favors the expansion of domestic artificial containers [8]. In addition to the optimum climate, other factors that increase the risk of transmission should be taken into account, such as the existence of suitable vegetation cover (bushy, shady areas) that serves as resting places for vectors, close to dwellings; anarchic urbanization (run-down housing, overcrowding) and poor sanitation (residual water sources, faulty water supply systems) that create larval breeding grounds [6]. *Ae. aegypti* is abundant in the peri-domestic environment, resting inside buildings on dark, damp walls and objects. Buildings are therefore the hunting grounds of these mosquitoes, which feed almost exclusively on human blood during the day and at dusk. Population density and human mobility can therefore influence dengue transmission [1,7,9]. In West Africa, a case of dengue fever was first reported in Nigeria in the 1960s. Since then, and particularly over the previous two decades, the four dengue serotypes have co-circulated in the region, resulting in sporadic cases and epidemic reemergence [10].

To date, there is no effective anti-viral treatment for dengue fever. Despite the existence of vaccines with partial immunological efficacy, forecasting possible dengue epidemics and vector control remain credible strategies for combating this disease. We can act swiftly and implement targeted interventions, but this requires us to take into account the local environmental and contextual factors that govern the dynamics of this disease. Sporadic cases of dengue fever have been reported in Burkina Faso since at least 2003. In 2016, there was a notable increase in the number of cases, with 1061 cases in August and November, but it was in the last quarter of 2017 that the highest number of cases was recorded, leading to the first declaration of a dengue epidemic in the country. The central region was the most affected in the country. The aim of the present study is to investigate the spatio-temporal dynamics of dengue during 2017 in the central region of Burkina Faso [10,11,12]. This region is the main dengue cluster in Burkina Faso.

## 2. Materials and Methods

### 2.1. Study Site

**Figure 1:**
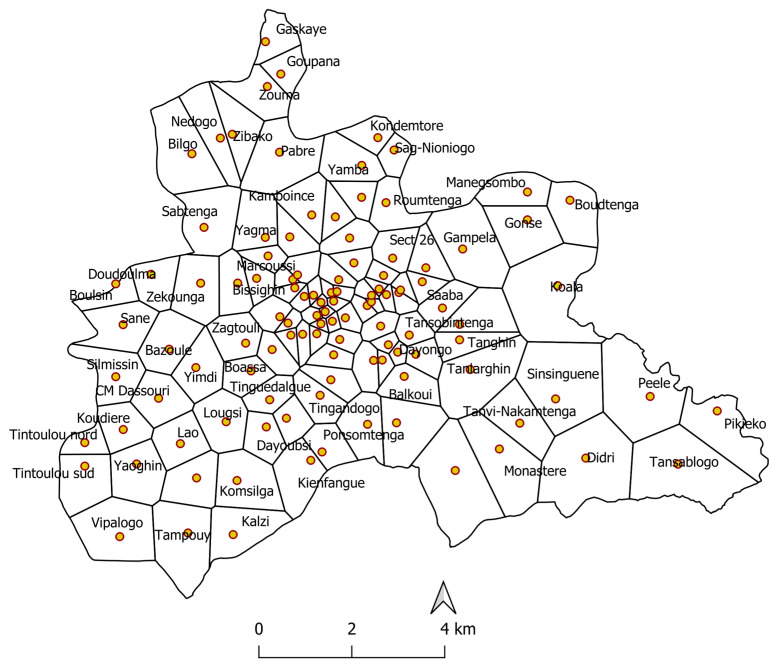
Boundaries of health areas (lines, Thiessen polygons) and location of health centers (dots) in the central region, Burkina Faso.

Burkina Faso is located in West Africa, bordered by Mali (to the north and west), Niger (to the east), Benin, Togo, Ghana and Côte d’Ivoire (to the south). The climate is Sahelian in the northern part and Sudanese in the rest of the country with two main seasons: the dry season is from November to May, when the harmattan, a hot wind, blows and temperatures vary greatly between day and night. The rainy season extends from June to October. The central region of Burkina Faso covers an area of 2869 km^2^, and it includes the urban commune of Ouagadougou (capital) and 6 semi-urban or rural communes. Its population was estimated at 2,744,666 in 2017, making it the country’s most populous region. The central region is subdivided into five health districts with 111 health centers (Figure 1). For each health center, the Ministry of Health defines an associated administrative health area. The health area is our spatial unit [13].

### 2.2. Description of Data

For this study, we used dengue cases reported to the Burkina Faso Ministry of Health in 2017. These cases were reported weekly by each health center, verified and compiled at the level of their health district of belonging, and transmitted to the Ministry of Health in accordance with the circuit of the national epidemiological surveillance system, the Weekly Official Letter Telegram.

The geographical coordinates of the various health areas extracted from the health map were provided by the Directory of Health System Information.

Previous studies in the central region had highlighted the irrelevance of associating cases of illness received in health centers with the corresponding administrative health areas. In fact, local residents prefer the nearest health facility rather than the one administratively associated with their home. Geographical coordinates were used to create point entities corresponding to health centers, and between these points, bisectors were drawn to delimit Thiessen polygons, which are theoretical health areas associated with health centers. In this approach, any location in a polygon is closer to its associated point than to any other input point entity.

The estimated populations per health area derived from population projections made by the National Institute of Statistics and Demography were extracted from the 2017 annual action plans of the various health facilities.

From a raster downloaded from the National Aeronautics and Space Administration’s remote sensing database (https://giovanni.gsfc.nasa.gov/ (accessed on 24 June 2020)), we extracted meteorological and environmental data using QGIS software. We thus obtained the following variables (Table 1):

- Weekly cumulative rainfall (mm);
- Weekly averages of daily minimum and maximum temperatures (°C);
- Weekly averages of daily minimum and maximum relative humidity (%);
- Monthly Normalized Difference Vegetation Index (NDVI);
- Weekly averages of daily wind speed (m/s).

We extracted the road network and land use from the openstreetmap database (http://download.geofabrik.de/ (accessed on 24 June 2020)). The length of each road was measured on QGIS, and their sums were aggregated by health area and road type (main, residential, and unclassified). The sum of vegetation cover areas was also calculated and aggregated by type (forest, farm, orchard and scrub) and by health area. These data were related to the surface area of each sanitary area to obtain road density and vegetation cover density. All data is available in supplementary materials.

### 2.3. Data Analysis

The cumulative incidence of dengue was calculated from the weekly number of cases reported in 2017 in the central region of Burkina Faso and at the health area level for the period of the reported epidemic.

First, meteorological and environmental factors were subjected to principal component analysis (PCA) to collinearities. The stationary of the time series derived from this analysis and the time series of dengue cases were determined with a Box–Jenkins ARIMA-type modeling procedure and their lags using the cross-correlation function [14,15,16].

Next, a generalized additive model (GAM) assessed the impact of meteorological and environmental factors on dengue incidence. In this model, principal components were included after stationarization and taking into account the time lag between series. A negative binomial distribution was used to account for overdispersion, and the logarithm of the population was used to estimate standardized incidence ratios [17]. The existence of spatial autocorrelation of dengue cases was assessed using the Global Moran I statistic, which measures whether the risk of dengue occurrence is constant across the central region.

High-risk health areas were identified using Kulldorff spatial scanning with Satscan version 9.4 software. This approach groups different neighboring spatial units into potential clusters by moving a scanning window across the geographical area of study. The algorithm uses circular windows centered on each health area. Potential clusters are defined for a radius ranging from 1% to 50% of the population [18].

Finally, we assessed the impact of land use (population density, road density and vegetation cover density) on the risk that a health area was at high risk of transmission over the reported epidemic period using another generalized additive model (GAM).

## 3. Results

### 3.1. Incidence of Dengue Fever

**Figure 2:**
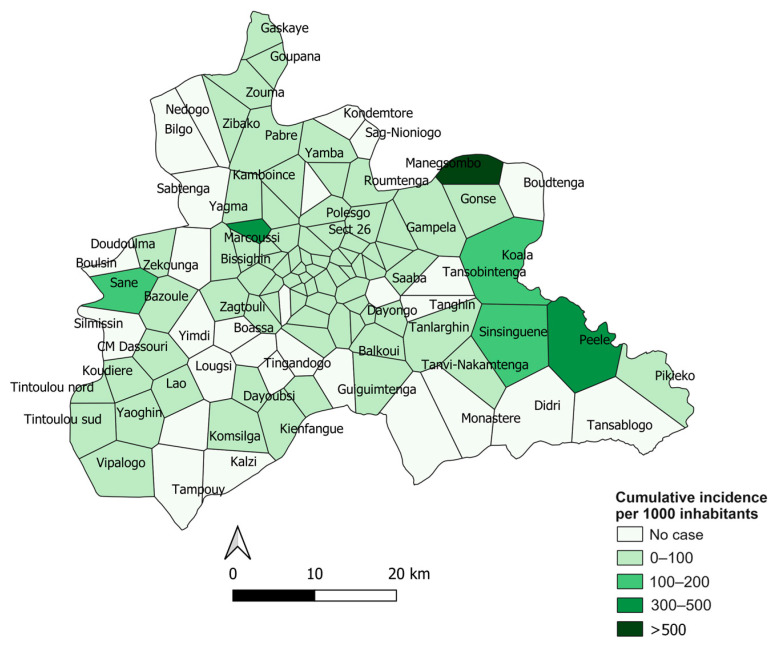
Incidence of dengue during the 2017 epidemic period by health area in the center region, Burkina Faso.

During 2017, 6894 cases of dengue fever were reported in the central region of Burkina Faso. The weekly incidence ranged from 0 to 52 cases per 100,000 people. The weekly incidence of dengue cases increased significantly in the last quarter of 2017. During this period, the country’s health authorities declared an epidemic in the region. Figure 2 illustrates this increase in incidence from week 37 onward, reaching a peak in week 43 (10-fold increase). The most affected health areas were Manegsombo, Peelé and Marcoussi (Figure 2).

### 3.2. Meteorological, Environmental and Dengue Incidence Data

**Figure 3:**
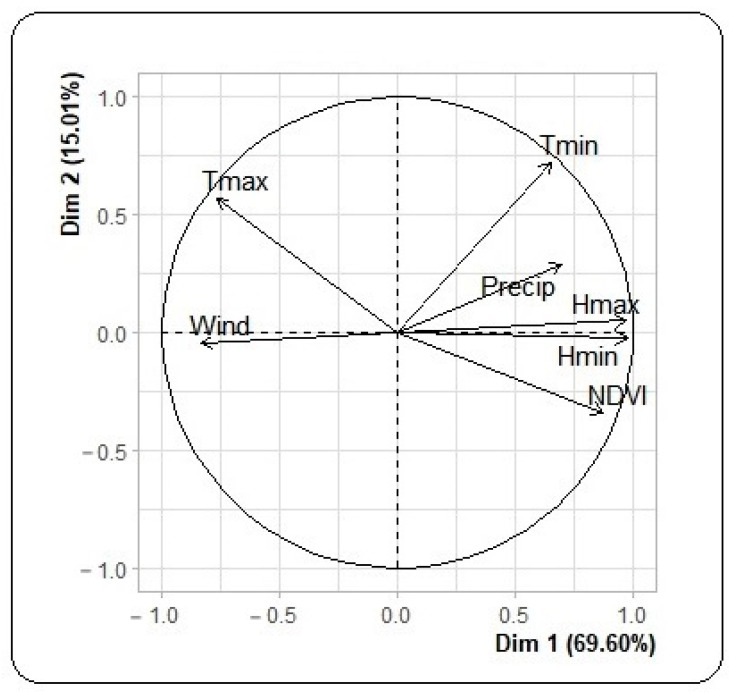
Principal component plot of meteorological and environmental factors for the central region in 2017, Burkina Faso.

A principal component analysis of meteorological and environmental factors identified two main axes representing 84.61% inertia (Figure 3). The first axis is made up of precipitation, relative humidity, vegetation index, wind speed and temperature. Minimum and maximum temperatures, precipitation and vegetation index formed the second axis.

After stationing, the first axis was positively and significantly correlated with dengue incidence (correlation coefficient: 0.7) with a lag of 8 weeks. The second axis was negatively and significantly correlated with dengue incidence (correlation coefficient: −0.3).

Multivariate analysis (GAM modeling) assessed the relationship between dengue incidence and the two meteorological principal components (taking into account the time lag between them). This model explained 83% of the variation in incidence and showed a significant relationship between dengue incidence and the two meteorological principal components.

**Figure 4:**
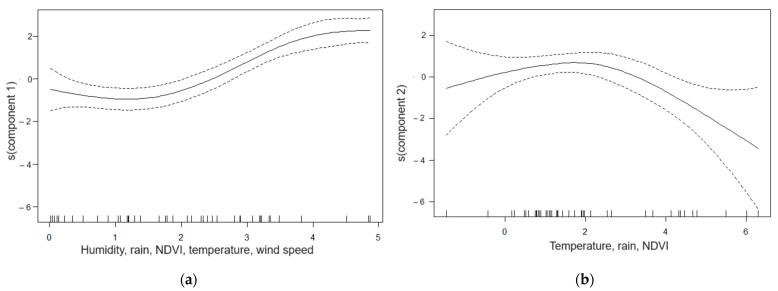
Relationship between dengue incidence and principal components. The solid curves represent the smoothing of dengue incidence according to the first principal component (**a**) and the second (**b**) with the 95% confidence interval in the dashed curves.

The relationship with the first principal component was positive and linear (*p* < 0.001; Figure 4a). Thus, dengue incidence increased with increasing maximum and minimum relative humidity, precipitation, normalized difference vegetation index, and minimum temperature but decreased maximum temperature and wind speed.

The relationship with the second component was two-phase (*p* < 0.001; Figure 4b). A first increasing part indicates an increase in dengue incidence with increasing minimum and maximum temperatures and precipitation and a decrease in the normalized difference vegetation index. A second decreasing section showing the opposite effect of high temperatures and precipitation and the absence of vegetation.

### 3.3. Spatial Distribution

**Figure 5:**
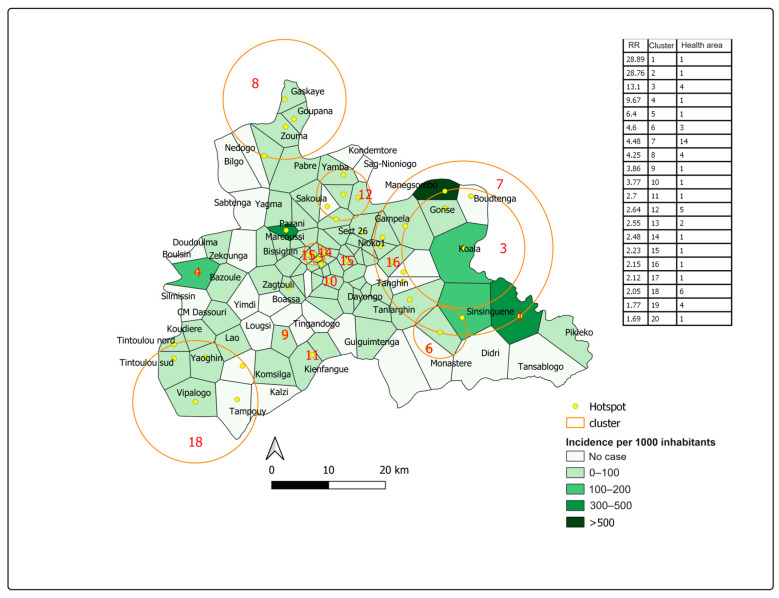
Dengue incidence by health area and spatial distribution of clusters during the 2017 declared epidemic period in the central region, Burkina Faso.

Spatial analysis showed a significant positive spatial autocorrelation of dengue incidence over the 2017 declared epidemic period in the central region, with a global Moran’s I value of 0.007 (*p* < 0.05). This means that health areas close to each other tend to have baseline incidence rates and that clustering patterns existed during this epidemic. We thus detected 20 high-risk clusters comprising 48 health areas (Figure 5).

The two highest-risk clusters had a relative risk of 28.89 and 28.76 (*p* < 0.0001) and comprised the Peelé and Marcoussi health areas, respectively, with a cumulative incidence of 328 and 340 cases per 10,000 inhabitants. 

The largest cluster was located in the southwestern part of the region. It comprised 14 health areas, including 10 in the rural commune of Saaba and 4 in the rural commune of Koubri. The relative risk was 4.48 (*p* < 0.0001) and the cumulative incidence was 45.3 cases per 10,000 people with a population of 145,123.

**Figure 6:**
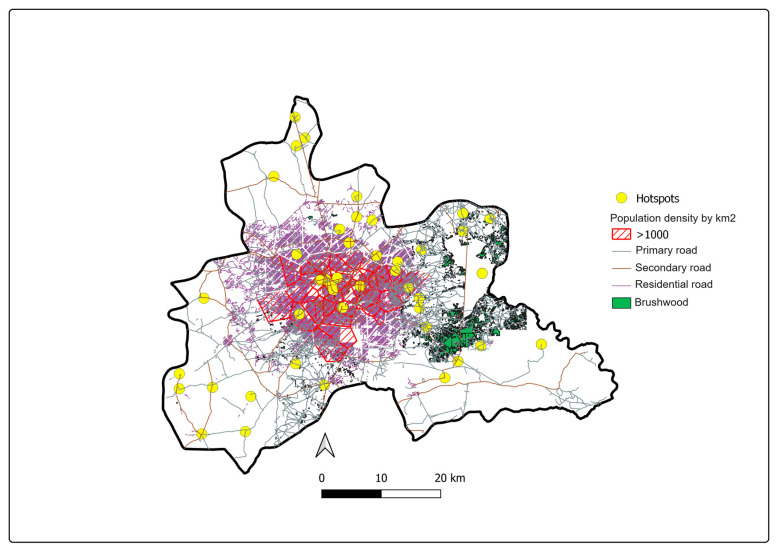
Land use and high-risk clusters during the epidemic period declared in 2017 in the central region, Burkina Faso.

The population density and the area occupied by brush were significantly associated with the risk of a sanitary area being at high risk. However, after adjustment between them, only scrubland areas remained significantly associated with this risk (Figure 6, *p* < 0.001).

## 4. Discussion

This study highlighted the spatio-temporal heterogeneity of dengue incidence in the central region of Burkina Faso.

The quasi-linear positive relationship between the first meteorological component and dengue incidence is similar to that observed in other studies [19,20,21,22].

For example, Hii et al. reported an increasing linear relationship between the relative risk of dengue fever and cumulative weekly rainfall in Singapore with a lag of 5 to 8 weeks. However, their study also showed that the effect of precipitation varied not only according to the lag but also according to the amount of precipitation [22]. These results are in line with the influence of precipitation on the breeding sites and egg hatching of Aedes mosquitoes described in the literature [23].

In this study, we found an 8-week lag between dengue incidence and the first meteorological component. This could be due to a combination of meteorological factors.

Positive correlations of dengue incidence have also been described with relative humidity [19] (two-week lag in the epidemic period [21]) and a differentiated normal vegetation index [20]. The dual impact of temperature on dengue incidence has also been demonstrated in several studies [24,25,26,27,28]. At optimal average temperatures (<18 °C), an increase in temperature increases the incidence of dengue fever by shortening the development period of *Ae. aegypti* larvae and improving their blood supply and oviposition [24], whereas at high temperatures, this increase will reduce the survival of Aedes and hence dengue transmission [24,25,26,27].

Detecting high-risk areas enables dengue control strategies to be focused, making them more efficient and cost-effective for countries like Burkina Faso, where resources are limited. Areas at high risk of dengue during the 2017 declared epidemic period were mainly the rural communes of Sabaa and Koubri and the Marcoussis health area. The weekly distribution of risk was relatively stable. What these areas have in common is that they are subject to uncontrolled urbanization. Marcoussis, for example, was created in 2006 by the hasty subdivision of part of the village of Bissighin in an emergency to cope with flooding in the area and the presence of Burkinabe refugees from the political crisis in Côte d’Ivoire at the time. The village of Peelé in the department of Koubri only had its first water borehole in 2014, and the department of Sabaa is still divided between urban activities, market gardening and pastoral activities. These areas are therefore confronted with problems of drinking water supply, sanitation and housing, all of which make them favorable breeding grounds for *Ae. aegypti*.

In fact, areas at high risk of transmission were significantly more overgrown with scrub, and the Aedes’ resting places were close to dwellings. In addition to these factors, there is the influence of human–vector contact, which is determined by the non-avoidance of mosquito breeding sites, and the ability of humans to create artificial mosquito habitats in their immediate environment. These behavioral factors were not included in the present study.

While it has been described that the central region of Burkina Faso is particularly high risk for dengue transmission [29], we report in this study that within the region, the spatial distribution is heterogeneous. This provides additional information for targeted control actions and demonstrates the importance of spatial unity in risk assessment. Furthermore, factors such as population density, road density and vegetation have not been taken into account in previous studies [30]. Taking them into account should enable us to improve predictive models of dengue incidence in Burkina Faso.

## 5. Conclusions

We analyzed the spatio-temporal dynamics of the 2017 dengue epidemic in the central region of Burkina Faso at smaller spatial scales. The results showed, on the one hand, a strong influence of meteorological factors on the distribution of dengue and, on the other hand, that this distribution was strongly correlated in space. The southwestern part of the region was most at risk. This identified spatio-temporal heterogeneity provides useful information for public health authorities to implement targeted dengue control strategies.

## Figures and Tables

**Table 1:** Data sources.

Data	Temporal Unit	Spatial Unit	Source
Dengue case	Week	Health area	Ministry of Health
Population	Year	Health area	Ministry of Health
Geographic coordinates and area	Year	Health area	Ministry of Health
Average wind speed	Week	Central region	NASA ^1^ https://giovanni.gsfc.nasa.gov/ (accessed on 24 June 2020)
Cumulative rainfall	Week	Central region	
Average minimum and maximum temperature	Week	Central region	
Average minimum and maximum relative humidity	Week	Central region	
Normalized Difference Vegetation Index	Month	Central region	
Population density (inhabitants/km^2^)	Year	Health area	
Road density (km/km^2^)	Year	Health area	OpenStreetMap (http://download.geofabrik.de/ (accessed on 24 June 2020))
Density of vegetation cover (km^2^/km^2^)	Year	Health area	

^1^ National Aeronautics and Space Administration.

## Data Availability

The data presented in this study are available in the Supplementary Materials.

## Supplementary Materials

The following supporting information can be downloaded at https://www.mdpi.com/article/10.3390/tropicalmed8110482/s1, Sheet 1: cases of dengue by health area in Central region of Burkina Faso; Sheet 2: Climate parameters by health area in Central region of Burkina Faso; Sheet 3: Dengue cases in Central region of Burkina Faso general additive model data.
